# Parental emotional, social and transitional health in the first 6 months after childhood critical illness: A longitudinal qualitative study

**DOI:** 10.1111/jan.16288

**Published:** 2024-06-24

**Authors:** Pei‐Fen Poh, Matthew C. Carey, Joseph C. Manning, Jan Hau Lee, Jos M. Latour

**Affiliations:** ^1^ Division of Nursing KK Women's and Children's Hospital Singapore Singapore; ^2^ School of Nursing and Midwifery, Faculty of Health University of Plymouth Plymouth UK; ^3^ Paediatrics Academic Clinical Programme Duke‐NUS Medical School Singapore Singapore; ^4^ Nottingham Children's Hospital Nottingham University Hospitals NHS Trust Nottingham UK; ^5^ School of Healthcare, College of Life Sciences University of Leicester Leicester UK; ^6^ Children's Intensive Care Unit KK Women's and Children's Hospital Singapore Singapore; ^7^ Department of Nursing, Zhongshan Hospital Fudan University Shanghai China; ^8^ School of Nursing Fudan University Shanghai China; ^9^ Present address: Drake Circus Plymouth UK; ^10^ Present address: Queen's Medical Centre Nottingham UK

**Keywords:** children, emotional outcomes, family, longitudinal follow‐up, paediatric, paediatric intensive care, PICS‐p, post‐intensive care syndrome, psychological outcomes, social outcomes

## Abstract

**Aim:**

To explore the experiences and support needs of parents in the first 6 months after paediatric critical care.

**Design:**

Longitudinal qualitative design.

**Methods:**

Sequential semi‐structured qualitative interviews were conducted with a sample of 28 parents in succession at 1 month and at 6 months (*n* = 22) after their child's discharge from paediatric critical care using purposive sampling. Data were analysed using the adapted five‐stage framework analysis.

**Results:**

Data were developed into eight synthesized themes, three domains and an overarching theme: Regaining Normalcy. Families of children requiring medical treatment at 6 months showed signs of adaption to daily care routines. The two domains were Parental Emotional Health and Parental Social Health. Parental Transitional Health, a third domain, was added to the Post Intensive Care Syndrome‐paediatric framework. Parents were forward‐looking and discussed emotional health, relating to current caregiving issues. Emotional attention was related to present challenges and concerns about current health and possible readmission to the hospital. In terms of Parental Social Health, families isolated themselves for infection control while remaining connected with families using chat applications. Parents were selective to whom they allowed access to their lives. The impact of parental transitional health was evident and emphasized the daily challenges associated with integration back to home life. Flexible work arrangements allowed working parents to support caregiving needs in the first 6 months after discharge.

**Conclusion:**

In the first 6 months after paediatric critical illness, most families reported having moved past the experiences while having provoking memories of the admission period. Parents viewed the point of normalcy as child returned to school or when all medications were discontinued. Extension of transitional support can facilitate discharge experiences between paediatric critical care and normalcy. The findings highlight the importance of understanding the medium‐ and longer‐term impact of paediatric critical care.

**Impact:**

- What problem did the study address?
  ○ Limited understanding of long‐term parental experiences and support needs after PICU discharge.
- What were the main findings?
  ○ Most families regained normalcy when child returns to school or when medications were discontinued. Some families continued to show signs of adaptations at 6 months after PICU discharge.
- Where and on whom did the research have an impact?
  ○ The research has an impact on improving the understanding of long‐term parental experiences and support needs after PICU discharge, informing clinical practice, guiding policy development and shaping parental support programs.

**Reporting Method:**

We reported this study using the COREQ guidelines.

**Patient or Public Contribution:**

Prior to confirming the interview guide, three parents of critically ill children actively participated by reviewing and providing feedback on its content. They provided suggestions to refine the wording and ensure clarity to enhance the participants' understanding. By including the perspectives of these parents, we aimed to improve the overall quality and relevance of the interview guide.

## 1 INTRODUCTION

In the evolving landscape of paediatric critical care in Singapore, as in other parts of Asia, recent studies have shed light on specific aspects of the post‐PICU challenges. However, a deeper understanding, particularly of parental experiences, remains poorly studied. A retrospective study conducted in a tertiary paediatric hospital in Singapore found that 8% of the PICU survivors acquired new morbidity after discharge, leaving an open question regarding the broader psychosocial impact on families (Senna et al., 2020). Complementing this, another study focusing on critically ill children with congenital heart diseases, highlighted significant stress and coping challenges faced by parents after PICU discharge (Poh et al., 2020). Research prioritization exercises in various regions of the world highlighted the importance of research needs in understanding the long‐term experiences of parents after discharge from the PICU (Poh, Sng, et al., 2022; Ramelet et al., 2012; Tume et al., 2014, 2019). Following discharge, up to 30% of PICU survivors' parents reported psychological symptoms such as post‐traumatic stress, depression and anxiety (Abela et al., 2020; Ko et al., 2022; Manning et al., 2017; Poh, Carey, et al., 2022). Perceptions and adaptations of parents are important for successful home reintegration after childhood critical illness (Chan et al., 2019; Foster et al., 2019; Williams et al., 2018).

## 2 BACKGROUND

The Post‐Intensive Care Syndrome‐paediatric (PICS‐p) framework illustrates the health domains that can be affected by critical illness, considering the interdependence of the child and the family unit (Manning et al., 2018). Following critical illness, parents may experience changes emotionally and socially. The emotional health domain encompasses the different impacts on the child's and family's emotional state after critical illness. The social health domain recognizes the impact of critical illness on the child's and family's social functioning such as interaction with peers and social networks (Manning et al., 2018). A recent mixed‐method systematic review showed that parents continue to experience emotional burdens, years after their children's PICU admission. In addition, support from the acute hospital was fragmented and families had difficulty getting paediatric support in the community (Rennick et al., 2021).

Current literature exploring the long‐term experiences of parents after PICU is limited in its consideration of the PICS‐p framework. To our knowledge, no studies have conducted repeated interviews with the same group of participants after discharge from the PICU. Understanding the trajectory of parental experiences following discharge may facilitate timely provision of support. The Singapore Health Outcomes After Critical Care Illness in Kids (SHACK) study is a mixed‐method prospective cohort study to examine children and their parents' health outcomes, 6 months after their hospitalization in the PICU (Poh et al., 2021). This paper reports on the qualitative component, to explore the experiences and support needs of parents in the first 6 months after discharge from the PICU.

## 3 THE STUDY

### 3.1 Aim

The aim of the study was to explore long‐term experiences of parents in the first 6 months after PICU discharge.

### 3.2 Research questions

What are the longitudinal experiences and support needs of parents up to 6 months after their child's PICU discharge?

### 3.3 Design

This paper presents the findings of the qualitative study. The quantitative study of the larger observational cohort study utilized serial self‐reporting instruments, guided by the PICS‐p conceptual framework at five time‐points. An embedded qualitative study design was chosen to explore the contextual, unique nature and evolution of parental experiences after discharge from the PICU. We reported this study in accordance with the Consolidated Criteria for Reporting Qualitative Research (COREQ) guidelines (Data S1) (Tong et al., 2007).

### 3.4 Theoretical framework

The PICS‐p conceptual framework was used to guide the development of the study design (Manning et al., 2018). PICS‐p operates on the premise that the parental journey through a child's critical illness is an active and interconnected experience. Parents undergo a complex emotional and social transition, marked by stressors, coping mechanisms and potential long‐term psychological impacts.

### 3.5 Study setting and recruitment

In the larger study, convenience sampling was used to recruit eligible participants (Poh et al., 2021). We recruited participants from a PICU in a tertiary paediatric hospital in Singapore. The study site is an 800 bedded hospital, specializing in child and women's health, with 400 beds dedicated to paediatric services, including a 16 bedded multidisciplinary (medical, surgical, cardiac and oncology) quaternary PICU facility. Recruitment was conducted by a researcher who, being an employee with unit access, did not provide direct care to potential participants to avoid conflicts of interest. A clinical research coordinator facilitated participant recruitment when the researcher was not available, 3 declined out of 31 eligible participants. Purposive sampling of participants was used to select parents of children representing a variety of diagnoses from the larger study (Polit & Beck, 2020). The interviews aimed to explore the experiences of at least 12 parents in the first 6 months after discharge from the PICU.

### 3.6 Inclusion and exclusion criteria

We included parents of children aged 1 month–18 years who were admitted to the PICU for a minimum of 48 h. Parents of children who received palliative services or had a non‐resuscitation order were excluded.

### 3.7 Ethical considerations

This research protocol was approved by the SingHealth Centralised Institutional Review Board (CIRB Ref: 2020/2997). Potential harm such as feelings of distress from reliving experiences were highlighted in the information sheet, with counselling helpline numbers provided to participants. Informed and written consent were obtained from the participants. Participants' names were replaced by child's gender, age and pseudo‐names were used for reporting. To ensure confidentiality, all data files were password protected.

### 3.8 Data collection

Data collection occurred from February 2021 to February 2023 using face‐to‐face or virtual semi‐structured interviews, conducted by the researcher (PF), for individual parents or paired parents. Interviews were conducted face‐to‐face or online, depending on participants’ preferences and prevailing COVID‐19 restrictions. An interview guide was used to elicit participant's perceptions of their experiences and support needs of the parents (Table 1, Interview guide). Probing questions promoted the clarification of their responses and the elaboration of recollections. Repeated interviews were conducted with the same group of parents 1 and 6 months after the child's discharge from the PICU. All interviews were audio‐recorded, pseudonymized and transcribed verbatim by the researcher (PF). Throughout the data collection process, we monitored for data saturation, this was achieved after the 20th interview. We conducted three more interviews to ensure that the data truly represent a comprehensive understanding of parental experiences after PICU discharge.

**TABLE 1:** Interview questions.

Interview questions	Probe(s)
It has been 1/6 months since your child was discharged from the PICU. Can you please tell me about how your life has been since the discharge?	What has been challenging?
How similar or different would you say your life have been compared to the time before your child was admitted to the PICU?	What was unexpected?
With the evolving COVID‐19 situation, how do you think these restrictions have affected your life after your child's PICU discharge.^a^	How have your activities changed?
Can you please tell me about the support you have received?	What kind of support did you feel was lacking?
What support did you wished you would have received?	
Can you please tell me how you feel as the primary caregiver of your child after the PICU discharge?	What would make a good/bad day for you?
Can you please tell me what you do to help you cope with the care giving needs?	How do you feel about your coping methods?
How has your interaction with friends and family changed since your child was discharged from the PICU?	How do you feel about these changes?
Has there been any other changes in other aspects of their life since your son/daughter left the PICU 1/6 months ago?	
Is there anything else we did not discuss and you want to share with me?	

^a^ Questions regarding COVID‐19 restrictions were added after three interviews when it became apparent, based on parents' responses, that this was a relevant area to be explored.

Our interview guide was developed, drawing upon seminal works that explore post‐PICU experiences, including Manning et al. (2018), discussions on the PICS‐p framework, Foster et al. (2019) and Williams et al. (2018) studies on parental experiences during and after PICU discharge. Three parents were consulted and provided feedback on the wording of the interview guide to improve understanding and usability. The interview guide was iteratively adapted after each interview by deleting, adding or fine‐tuning questions. All interviews started with a preliminary question about the current health of the child survivor. Subsequently, more in‐depth questions were asked to further explore thoughts, feelings and support needs. Baseline and clinical characteristics such as demographics (e.g. age, gender, education, religion and ethnicity), socioeconomic data and current admission data were collected. Clinical data were extrapolated from the electronic medical records. During data collection, it was noted that children were present at the time of the interviews in some instances. This was reflective of the familial environment after critical illness, where parents often cared for their recovering children at home. Each interview was conducted in a way that respected the family's dynamics, ensuring parents felt comfortable to speak freely, whether or not their child was present.

### 3.9 Data analysis

Audio‐recorded interviews were conducted and transcribed verbatim by PF with field notes incorporated in brackets. Interviews were conducted in English, the primary language of business and healthcare in Singapore; no translation was required. Interview data were analysed using the a priori framework based on the domains of the ‐PICS‐p (Manning et al., 2018) which posits that the parent social and emotional health are interdependent with their children's PICU admission and changes overtime. Two members of the study team (PF, MC) read the transcripts and conducted line‐by‐line coding to describe key components of the data. Coding of the first five interviews was returned to the study team for checking (JL, JM JH). NVivo 12 (released in March 2020) software was used to manage the interview data and to enhance the study rigour (Polit & Beck, 2020).

Data collection and analysis were iteratively approached, and it became apparent after the initial interviews that COVID‐19 was significant to parents' experiences during PICU admission and following discharge. Although we did not set out to assess the impact of COVID‐19 on post‐PICU experiences, it was clear that the parents drew links between the isolation measures, changes in hospital restrictions and work arrangement. These were confirmed and explored in subsequent interviews. A decision was made by the team to include the impact of COVID‐19 on their recovery, as it emerged as a pervasive theme across all interviews.

Analysis with the a priori framework based on the domains of the PICS in paediatrics was used. The adapted five‐stage framework analysis was used to examine the experiences and support needs of parents after their child's PICU discharge (Ward et al., 2013). Initially, we used open coding to identify preliminary themes that were directly derived in the data (Appendices S1 and S2). Subsequently, mapping themes helped us to relate these themes to the broader theoretical constructs of the PICS‐p framework. Selective coding was then engaged, where we systematically refined and integrated these themes to develop a comprehensive analytical framework (Appendix S3). This framework was informed by and expanded upon the a priori theoretical constructs, incorporating emergent themes such as ‘financial impact of caregiving’ and ‘parental role differences in care provision’, which were not initially part of the PICS‐p framework. These themes recurred across interviews and were considered significant to the parents' experiences; hence, a third domain was adapted into the original PICS‐p conceptual framework.

### 3.10 Rigour and reflexivity

The data analysis was conducted collaboratively by PF and the other co‐authors to assess credibility, originality, resonance and usefulness, ensuring alignment and relevance (Saldaña, 2016). Rigour was maintained through immersion of the data (Ward et al., 2013). Credibility and resonance of interpretations were strengthened through participant feedback regarding findings and interpretations along with a detailed and comprehensive description of the data (Saldaña, 2016). Reflective field notes were taken to capture the gestures and reactions as the participants unveiled their emotions. The research team (JL, JM, MC and JH) provided feedback on the first three interviews and provided guidance on the use of prompts to elaborate on potential points of interest. The interviewer conducted a check with the study participants during data collection to validate data interpretation and to guide subsequent interviews (Polit & Beck, 2020). In addition, the interviewer who was a female, Singaporean, Chinese PhD student and a paediatric critical care nurse, recorded her reflections on each interview to capture the interviewer's perception and researcher bias towards the data collected. The use of researcher reflections served as an important tool for ensuring the quality and rigor of the qualitative data (Polit & Beck, 2020).

Multiple strategies were employed to establish trustworthiness of the study findings (Lincholn & Guba, 1985). Credibility was enhanced by including parents of children with a variety of admission diagnoses and clinical trajectory during and after critical care admission. During data collection, members checks were conducted with study participants to validate data interpretation, with no request for changes were made to the interpreted data (Saldaña, 2016). Triangulation of interview transcripts, field notes and multiple deliberations among study members contributed to the confirmability and credibility of the study finding (Lincholn & Guba, 1985).

## 4 FINDINGS

### 4.1 Characteristics of participants

Parents of 23 PICU survivors with different medical diagnosis and critical illness trajectory, completed an interview between 1 and 2 months post‐PICU discharge. Of which 22 parents completed the second interview between 6 and 7 months post‐discharge (Tables 2 and 3). At 1 month following discharge, of the 28 parents interviewed, participants included 13 mothers, five fathers and five pairs of parents. Twenty‐one interviews were conducted over video calls, two interviews were conducted face‐to‐face in the hospital compound. The average interview length was 23 min (9–76 min) at 1 month and 28 min (7–43 min) at 6 months.

**TABLE 2:** Characteristics of child participants (*N* = 23).

Characteristics	Median (interquartile range)
Age^a^, years	8 (2.5–11.5)
Male	15
Ethnicity	
Chinese	9
Malay	8
Indian	5
Others, Israeli	1
Length of PICU admission (days)	2 (2–4)
Length of Hospitalization (days)	16 (7–22.5)
Diagnosis	
Medical	8
Cow's milk allergy	1
Diabetic ketoacidosis	1
MIS‐C (COVID‐19)	2
Myocarditis (ECMO)	2
Renal failure (CRRT)	1
Respiratory failure background of cerebral palsy	1
Surgical (non‐cardiac)	3
Fall injury	1
Motor vehicle injury	1
Thyroid tumour resection	1
Cardiology	7
Atrial septal defect repair	3
Congenital heart block	1
Ventricular septal defect repair	3
Oncology	5
Brain tumour	1
Leukaemia chemotherapy	1
Leukaemia‐related and pneumocystis pneumonia	1
Neuroblastoma	2

Abbreviations: CRRT, continuous renal replacement therapy; ECMO, extracorporeal membrane oxygenation; MIC‐C, multisystem inflammatory syndrome in children; PICU, paediatric intensive care unit.

^a^ Range = 2 months–18 years.

**TABLE 3:** Parent participant characteristics (*N* = 23).

Characteristics	Median (interquartile range)
Age^a^, years	40 (36.5–42.5)
Father	7 (30%)
Mother	16 (70%)
Ethnicity	
Chinese	9 (40%)
Malay	8 (35%)
Indian	5 (22%)
Others, Israeli	1 (3%)
Religion	
Buddhism	5 (22%)
Christianity	1 (4%)
Hinduism	3 (13%)
Islam	10 (44%)
Jewish	1 (4%)
No religion	3 (13%)
Education	
Primary	2 (9%)
Secondary	4 (17%)
Polytechnic	6 (26%)
Undergraduate	4 (17%)
Post‐graduate	7 (31%)
Income (Singapore dollar)	
<1499	3 (13%)
1500–2999	3 (13%)
3000–5999	6 (26%)
6000–8999	8 (35%)
12,000–14,999	1 (4%)
>15,000	1 (4%)

^a^ Range = 31–75 years.

Six parents did not participate in the interview at 6 months post‐discharge; multiple attempts were made to reach out to parents who did not respond to the invitation for the second interview. Only one Malay mother cited that her work scheduled has changed to accommodate her child's multiple appointments and hence was unable to participate in the second interview. Among the six parents who did not engage in the second interview, three were mothers from the Malay community, no further exploration of the reason why was undertaken according to the study protocol.

### 4.2 Overall findings

**FIGURE 1:**
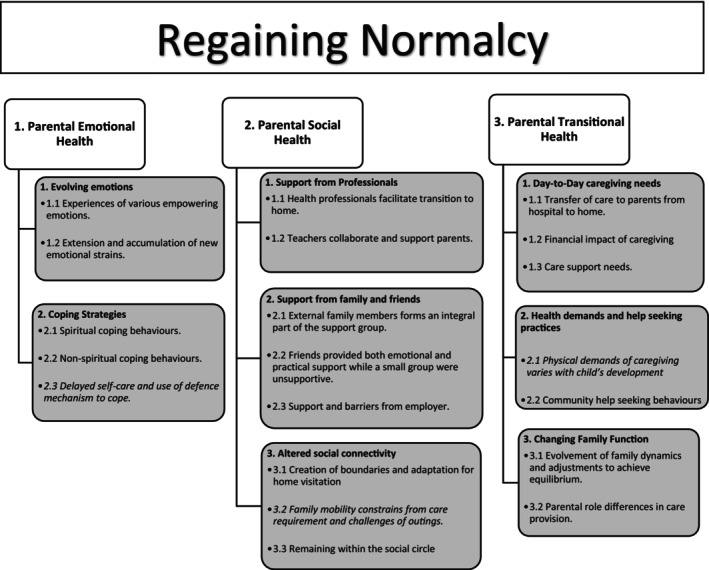
Domains, themes and subthemes of SHACK study qualitative interview at 1 month and 6 months post‐PICU discharge. *^1.2^Subtheme ‘Delayed self‐care and use of defence mechanism to cope’, ^2.3^Subtheme ‘Family mobility constrains from care requirement and challenges of outings with the use of medical devices’ and ^3.2^Subtheme ‘Physical demands of caregiving varies with child's development’ were not present at 6 months.

**FIGURE 2:**
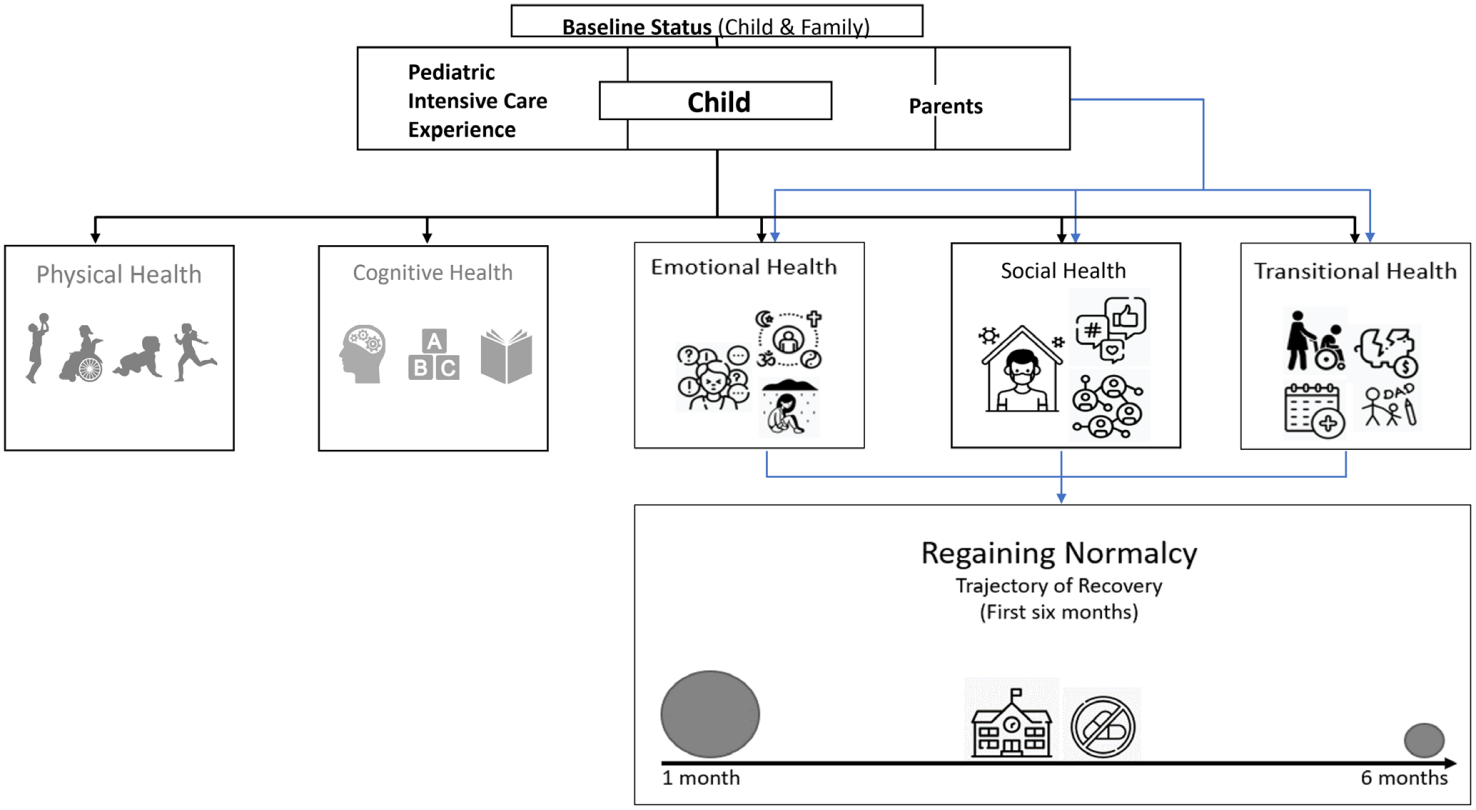
Parental experiences and support needs in the first 6 months after PICU discharge. Adapted from the Post Intensive Care Syndrome – paediatric (PICS‐p) framework.

Parents described emotional, social experiences and transitional efforts to readapt to life routines and integration of new care needs following PICU discharge. Regaining normalcy was identified as the overarching theme with data aggregated into 20 subthemes, eight synthesized themes and three major domains related to the PICS‐p framework: (1) parental emotional health, (2) parental social health and (3) parental transitional health (Figure 1). Participants recounted how the journey of their children in the first 6 months, following PICU discharge has impacted their daily lives. Their child's critical illness disrupted family routines. Participants felt that they generally coped by focusing on readaptation to normalcy while experiencing various challenges. Most families assume normalcy as the child returned to school. At 6 months, most parents of children reported that life has returned to normal. A small group of parents, whose children were suffering from cancer, congenital heart diseases or complications from the critical care admission, continued to show signs of adaptation to family routines. We expanded on the original –PICS‐p framework to include transitional health experiences of parents from this study (Figure 2).

### 4.3 Findings at 1 month

#### 4.3.1 Parental emotional health: Evolving emotions and coping strategies

Emotions evolved along with their child's PICU admission and home discharge journey. Parents reported feelings of gratitude and feeling overwhelmed from the critical illness, home integration and persistent worries over health deterioration, while in the community. Despite the evolvement of emotions, parents used various coping behaviours to adapt to the daily needs of their children.

Empowering emotions included gratitude over child's survival, improvement in health status, acceptance of the current state‐of‐health, overcoming own's fear and having trust in the health system. One mother described being in a conflicted state of emotional perturbation:

(I am) scared you know, I am to carry out injection at home, but I have to be brave for him, he is brave. (M4 Mother, aged 35 at 1 month)

While a small group of parents continued to question the cause of the critical illness, most parents have shown acceptance towards the PICU admission, medical diagnosis, child's condition and new medical care needs.

Emotional strains persisted following discharge, stemming from the critical care admission with cumulative stresses from the transition to step‐down hospital care and to home. Mother of a 2‐year‐old girl, described her deep‐seated anxiety about the potential readmission of her child to the PICU:

She was discharged, every night, even in the middle of the night, my hand, automatically will go and feel her heartbeat. The doctor told me that her heartbeat stopped once. (F3 Mother, aged 40 at 1 month)

Many parents turned to spiritual coping behaviours such as prayers, believing in God and the use of meditation and quiet time, as a source of relief. One mother highlighted her dependence on spiritual practices as a coping behaviour after her child's critical illness:

Prayer is important, it keeps the family bonded and there ah, the internal strength. (M14 Mother, aged 42 at 1 month)

Parents who perceived themselves as non‐religious coped by focusing on the reintegration of routines, participated in exercises, voluntary work and were motivated by daily successes of adaptations. One parent spoke of coping with their child's post‐PICU recovery by continuing with life's routine activities, embodying a practical, non‐spiritual approach:

Life goes on, but we know it's not the same, deep down, we know that is not the same (clear throat). (M11 Mother, aged 43 at 1 month)

Overwhelming care demands has resulted in delayed selfcare, while parents prioritized family and care needs of their children over self. One mother reflected on the postponement of her own self‐care needs, choosing to prioritize her family's needs during the challenging recovery period:

The focus right now is my work and my kids, but emotions (wise), actually, I always put myself aside (pause). (F3 Mother, aged 40 at 1 month)

In addition to the emotional strains, parents had to manage unpleasant encounters arising from their community. Some parents chose to avoid potential conflict, one mother shared her experiences of steering clear of conflicts with those who lack understanding of her child's illness and ongoing challenges:

It's very difficult for them (friends) to understand. I don't like judgement about Ethan, due to his medication (steroids), make him more puffy and bigger. (M2 Mother, aged 35, at 1 month)

#### 4.3.2 Parental social health: Support from professionals, support from family and friends and altered social connectivity

Parents recounted various forms of support they received following PICU discharge, as their child reintegrated back to home and school. Continuation of support from the healthcare systems emerged strongly in tandem with the help from family members. Following discharge, families remained supported by health professionals. External family members provided day‐to‐day support to the parents. In the first month of discharge, parents shared how they adapted to reduced physical socialization while remaining in touch with their social circle. All children were receiving outpatient follow‐ups with various medical specialists and allied health professionals at 1 month after discharge. School supported the child by providing concessions such as the use of school elevators, extended examination time and assigned buddy for assistance.

External family members were integral in providing support. Grandmothers emerged as the pinnacle in supporting parents. One mother recounted how she leaned on the support of her mother for assistance:

We rely on (my mother) and can help us with everything, especially I know for me and my mother here. (M11, Mother, aged 42 at 1 month)

Roles of the paid stay‐in help were expanded to support parents in the care of their child. New roles included monitoring of child's health, such as pain and blood pressure, and relief parents of medical duties such as nasal suctioning and insertion of the naso‐gastric tube. A parent discussed their dependence on her helper for managing complex home care tasks:

Bibik (paid stay‐in help) is taking effort (to) learn how to (perform nasal) suction, she was taught how to clean the CPAP (continuous positive airway pressure) mask and measure the NG (nasogastric) tube. (M4 months, Mother, aged 43 at 1 month)

Parents limited visitations and outings for infection control while remaining connected with families and friends through social media and group chats. Families of children who required mechanical ventilation and tube feeding reported that it was logistically challenging to get out of the house. A parent described the challenges they now face in taking their child out due to the new requirement for the non‐invasive ventilator:

We always bring him out last time, you know. now with the CPAP he is confined to home. Now if we bring him out, we need to bring a lot of things also. It's a very big thing for us. (M 9 Mother, aged 40 at 1 month)

#### 4.3.3 Parental transitional health: Day‐to‐day caregiving needs, health demands and help‐seeking practices and changing family function

Parents described similar experiences with transition towards home in the first month after PICU discharge. All children needed some form of physical support during the first 2 weeks at home. One mother shared her experiences of assisting her previously independent child with activities of daily living, driven by a fear of potential wound infection and falls.

We are concern over the wound and (when) she wants to wash her hair, we are afraid, just to make sure she is safe, don't want her to fall. (F7 Mother, aged 40 at 1 month)

Some parents assumed nursing tasks to provide monitoring and care while at home. Discussing home healthcare practices, one father detailed his use of hospital‐grade health monitoring, such as a stethoscope to closely monitor his child's condition:

We used the stethoscope to manually record it, the heartrate. (M2 months, Father, aged 38 at 1 month)

Additional care expectations contributed to accumulated feelings of fatigue in parents. Reflecting on the additional caregiving responsibilities, a parent described the exhaustion they experienced as a result of these heightened care demands:

I can see for myself, I come out from my body, I can see that I am not taking good care of myself. I just don't have any activities; I am just not in the mood to like make love or or beautify myself. (M 4 months, Mother, aged 43 at 1 month)

A mother hesitantly shared her struggle with feelings of guilt for viewing the care of her previously independent child as burdensome:

[Pause] at first I uhm … (taking care of him was) a heavy burden [pause] because it's not easy, especially if he don't have strength uhm because I will have to uhm, support him physically which uhm, Aish is already taller than me so uhm… I don't know (fast). (M14 Mother, aged 42, at 1 month)

Parents continued to seek help in the community and alternative medicine for a cure. One parent discussed exploring alternative medicine options to seek a cure for their child's condition:

We do believe in TCM (Traditional Chinese Medicine), the doctor said, there was no way she can convert the Baby Noah's condition to a normal rhythm heartbeat. So, we tried TCM. (M2 months, Father, aged 38 at 1 month)

Overall, parents reported closer relationship between spouses and child's siblings who have participated in the care of the child. However, a mother expressed her wish for greater involvement from her husband in the care of their child with medical needs at home:

Because we have a PICC (peripheral inserted central catheter) line and we also need to learn how to flush, I also curious how the parents can share the work, daddy do what, mummy do what. It's all on me. (M2 Mother, aged 35 at 1 month)

### 4.4 Findings at 6 months

#### 4.4.1 Parental emotional health: Evolving emotions and coping strategies

Intensity of emotional strains was lightened, and feelings of guilt, worries and uncertainty were related to current physical health. Parents continued to worry over potential readmission. Reminiscing of the admission evokes strong emotions such as terror and somatic symptoms. However, these intense feelings resolved overtime. Parenting sense of responsibilities emerged strongly as they recognized the need to overcome adversities to care for their child. One mother shared about her child who had suffered a road traffic accident with behavioural changes after PICU discharge:

Nah (laughter), I gave birth to her, so I have to bear with her bad behaviour. (F9 Mother, aged 43 at 6 months)

One parent conveyed a sense of accomplishment in successfully integrating their child's new need for suctioning, into their daily routine.

So far he is doing well, we have been coping with the (oral and nasal) suctioning and the daily routines we are getting used to the routine already. (M9 Mother, aged 40 at 6 months)

Parents remained hopeful as child approach milestones for completion of treatment or for medical discharge. Feelings of worry over possible acute health deterioration and uncertainty remained poignant as shared by one mother:

Not like we are discharged, and we will be okay… he cannot do the wrong (Insulin) calculation, if not he will need to go back to the ICU (intensive care unit). (M12 Mother, aged 39 at 6 months)

After experiencing critical illness, some children did not fully recover, instead acquired morbidities and have received life‐limiting diagnoses. Parents described their change in life perspective after the PICU admission:

I can say that our life can never be the same anymore… perspective now is a bit different, there Is like a shadow in our life all time, we live with it. (M11 Mother and father, aged 42 at 6 months)

Six months after PICU discharge, as children have reintegrated into school, some parents noted that their children were lagging academically. A parent shared their observations of their children struggling to keep up with schoolwork upon returning to school:

After the road traffic accident, she was not keen to learn because it was very obvious that her Chinese and Mathematics had deteriorated a lot. (F9 Mother, aged 42 at 6 months)

Some parents experienced flashbacks and somatic symptoms as they reviewed photos taken during the admission. A parent described vivid flashbacks of those intense and stressful days:

When I scroll through my photo gallery and see his photos, I will get flashbacks. We thought we might lose him, that kind of feelings. (M9 Mother, aged 40 at 6 months)

Parents continued to engage in prayers for health, focused on reintegration of family routines and engaged in exercises. However, some parents recounted avoiding memories of critical care admission and found themselves unable to talk about these experiences with their loved ones:

I rather not talk about it; I am scared that it will trigger the thing (panic attack) back. Even my husband doesn't know that I had sleepless nights. (F12 Mother, aged 39 at 6 months)

#### 4.4.2 Parental social health: Support from professionals, family and friends and altered social connectivity

Most children had returned to their baseline school activities while educators continued to provide supervision and modified activities for children who were on medical treatment. Families expanded their network and connected with families of children with similar experiences. Some grandmothers adopted a more permanent role, such as moving in with the family to provide continuous support. The paid stay‐in helpers have returned to their previous roles, as the children return to their routines. Most parents have returned to work, however, one mother reported frustrations over taking paid leave to attend to her child's medical appointment, noting:

erm… TSK. (frustrated) I do feel the stress and the impact of it, I kind of sometimes feel quite an unfair treatment (by employer). No exceptional handling and all that. (F12 Mother, aged 39 at 6 months)

#### 4.4.3 Parental transitional health: Day‐to‐day caregiving needs, health demands and help‐seeking practices and changing family function

Most families transitioned back to their previous routines. Parents of children who required new routines, such as insulin injection, peritoneal dialysis or suctioning, reported integration of new care into their activities of daily living. In addition, parents of children who were on active medical treatment continued to experience challenges relating to staying vigilant, unequal parental role distribution and support from employer. A mother spoke about the reality of frequent medical appointments, reflecting on how the hospital has become an integral part of their family's life:

The tumour was putting pressure on the eye nerve, they wanted to monitor…You know it's different, the hospital will be part of our life from now on. (M11 Mother, aged 42 at 6 months)

A mother candidly shared her experience of resuming night feedings for her teenage son, to fulfil the increased caloric requirement of her son undergoing chemotherapy:

That means I have to wake up at night, it's like back to baby day (laughter) the mother has to feed, he will need 2000 cal a day, the normal food that he eats does not add up to the calorie. (M15 Mother, aged 42 at 6 months)

Parents spoke about their heightened vigilance regarding any health changes in their child, ensuring to intervene early, to prevent a PICU readmission:

Yes, of course, every time he falls sick ah, me and my wife, we better do something before it gets worst. Once he don't drink milk, I already felt scared. (M1 month, Mother and father, aged 35 at 6 months)

Financial concerns emerged strongly for families of children requiring ongoing medical treatment and for mothers who have left the workforce to accommodate to caregiving needs. One mother shared that she has left her previous employer as they were not accommodative to her caregiving needs:

So! they indirectly tell me to leave by giving me another load of work of my ex‐colleague who has resigned. 90% of his job on top of my job and I was too stress everyday waking up crying, struggling and my (breast)milk also decreased (sobbing). (M 4 months, Mother, aged 43 at 6 months)

At 6 months, this mother was self‐employed, in a tone of resigned acceptance, she reflected on her financial hardships, stemming from extended work hours and income instability, noting:

Long trip (food delivery), there's a saying “beggar shouldn't be choosy” even though I am not a beggar. At least I can pay off my petrol even though I am sad, but yea (laugh). (M 4 months, Mother, aged 43 at 6 months)

### 4.5 Findings on trajectory from 1 to 6 months post discharge

#### 4.5.1 Parental emotional health: Towards emotional stabilization

Parents exhibited progression towards emotional stabilization with less report on emotional strains and more successful incorporation of new care needs and remaining hopeful for medical discharge from the hospital. Topics relating to sense of gratitude, relief and thankfulness over survival and acceptance of medical diagnosis were not mentioned at the 6 months. However, feelings of being overwhelmed at the remembrance of the critical care admission emerged for the first time at 6 months after discharge. One mother shared in a shaking voice, stress symptoms she experienced as she recalls her child's PICU admission during the initial post‐discharge, noting:

It just feels very overwhelming as if it is happening all over again and then I have added imagination like as if she is not going to wake up then I get teary. I (would) start shaking. (F12 Mother, aged 39 at 6 months)

The same parent also shared that the feelings were less intense over time, noting:

As time goes by, things will get better, the initial terror and the fear, and the sleepless nights that I had, it died down after the kid started getting better and better. (F12 Mother, aged 39 at 6 months)

Subthemes relating to delay of self‐care and the use of sarcasm as a protective mechanism did not emerge as part of coping at 6 months. Parents were also keen to move on from the paediatric critical care experience and to carry on with their normal lives, noting:

I mean it's okay, we are all back to normal. There is no sense in looking back and thinking about it you know. We are glad he is okay, that is the most important thing. Other than that, we just go about our normal lives, not thinking about it too much’. (M8 Father, aged 40 at 6 months)

#### 4.5.2 Parental social health: Towards social capital expansion

In the first 6 months, following paediatric critical care discharge, parents adjusted the way they socialize. Reasons for adjustments includes infection control, changes in family's mobility and they were selective towards friends they met. Parents were subjected to negative comments and intrusive questions relating to the cause of critical care admission and the morbidity their child has developed as a result of the critical illness. One mother shared her struggles receiving unhelpful comments from relatives, noting:

I know some of my families are trying to be helpful, but sometimes when they say certain things, they come across like asking questioning and asking why we are not doing well. You just felt that you are not a good parent. (M12 Mother, aged 43 at 1 month)

Although parents reported that they met friends less often, they had strong support from family and friends. During the initial phase of the critical care discharge, parents relied on formal support from the healthcare professionals and school teachers to reintegrate back into the child's routine. Overtime, parents expanded their network by meeting both local or international parents of children with similar experiences on social media or through word‐of‐mouth, where they felt the ease of connecting through shared experiences. One father spoke about the support he received from another parent of children with the same condition as his child:

I met one to two parents, face‐to‐face, there's one guy, he got 3 children, 2 of his children are having kidney problems. he pacifies me lah, He must have seen that I couldn't take it. (F12 Father, aged 75 at 6 months)

One mother shared her experience of connecting with overseas support group for children of the same condition as her son, requiring non‐invasive ventilator, after failing to locate a local support group:

Would be better if there is a support group for parent ya. So that they can share their experiences, like how they can cope. Currently, I don't see any lah, I found one in Facebook but that's overseas. (M9 Mother, aged 40 at 6 months)

While parents became more resourceful in medical knowledge and in help‐seeking, support from employers were short‐lived. Parents of children requiring medical care, experienced more difficulties in getting support for paid leave. Overall, parents experienced increased social capital in most areas of their lives while support from employer remained a challenge.

#### 4.5.3 Parental transitional health: Towards ownership and independence

At 6 months following PICU discharge, majority of the families have returned to their previous routines. Some families regained sense of normalcy within the first 3 months of discharge. Signs of normalcy included activities such as returning to school and discontinuation of medication. Relieved, a father expressed that life seemed to return to normal once all medications were no longer needed for his child, noting:

So for three months, we were actually more of caring on him, after that three months, we got the appointment and they ask us to stop the medications and we (laugh) were more relief. (M8 Father, aged 44 at 6 months)

Most children were able to perform their activities of daily living independently, requiring no additional support from their parents. However, parents of children with ongoing medical issues continued to experience exhaustion from long‐term caregiving needs. Some children were discharged with medical devices such as a peripherally inserted central catheter (PICC), peritoneal dialysis, mechanical ventilation, tracheostomy and insulin injection. Describing her family's caregiving structure, one mother shared that the care model has evolved, where she shouldered most of the child's care responsibilities, leading to her exhaustion while the father focused on being the sole breadwinner:

Most of the time I have to stay with him. Because now my husband is the only breadwinner. Seldom I get rest day, because on my husband's rest day, he will rest, and he won't help. Its only between me and my mum. But mostly let my mum rest. (M2 Mother, aged 35 at 6 months)

Diverse levels of fatherly engagement were seen. Although efforts were made to recruit fathers for participation, only seven consented. Of these, one father identified himself as the primary caregiver for the child. Regarding employment status post PICU discharge, three mothers left employment (M2, F12 and M14) and three transitioned to part‐time work (M4 month, M4 and M11) to accommodate to the medical needs of their children. Most mothers reported spousal involvement in the care of the children, fathers were more involved in play than caregiving which was in line with the general literature (Kazura, 2000). However, some mothers have wished for more paternal involvement in the medical care of the child.

Parenting role distribution at 1 month post‐critical care discharge shaped spousal relationships at 6 months. Discussing her family's approach to their son's complex care, one mother highlighted the importance of teamwork and family dynamics in their daily routine:

When my husband is back from work in the late afternoon, he will take over and I will get some nap. It's a family thing, my husband will step in to help after work. (M15 Mother, aged 42 at 6 months)

Sharing a contrasting experience, another parent voiced her frustration about her spouse lack of involvement in their child's care:

Sometimes we have conflict and usually they (fathers) won't help. Man, usually won't help. (M2 Mother, aged 35 at 6 months)

In summary, the diverse experiences shared by these parents underscore how family dynamic and parental role distribution shaped parental's perception of transition during the first 6 months after PICU discharge.

### 4.6 COVID‐19 restrictions on post‐intensive care recovery

The post‐discharge experiences of families were impacted by the COVID‐19 restrictions. Concerns regarding infection, including COVID‐19, have led parents to actively isolate themselves. Families were selective of visitors, preferring to remain in touch through social media and group chats. Discussing the impact of the pandemic, a mother revealed how her family self‐imposed restrictions post‐discharge to avoid COVID‐19 exposure, noting:

Because of the COVID, we sort of stay safe and control the visitors and her immunity is not that good so we just video call for the time being. (F12 Mother, aged 35 at 1 month)

Parents cited that the flexibility of a hybrid work arrangement according to the mandatory safe distancing restrictions, had facilitated home returning after critical illness. Although physically isolated, families described the various support they received during the first 6 months after discharge. Reflecting on the challenges posed by the pandemic, another parent recounted isolation as their relatives in Malaysia were inaccessible due to border closures:

We don't have any family members in Singapore. Just us. our families are all in Malaysia. we told my parents about Chris's condition. Because of the COVID‐19 they cannot come over. We are the only ones here; we are coping everything ourselves. (M12 Mother, aged 39 at 6 months)

## 5 DISUCSSION

In alignment of the PICS‐p framework, our study focused on parents experiences after their child's initial PICU admission. By concentrating on this initial admission, we delved into specific impacts of a child's first critical illness experiences on parental emotional, social and transitional health. This approach allows us to contribute empirical evidence to the PICS‐p framework, specifically examining how an initial PICU admission shapes parental experiences. The paediatric critical care experiences continue to have an impact on all the participants 1 month after discharge. Some participants whose children had unresolved medical issues and were receiving active medical treatments remained impacted at 6 months post‐discharge. Following discharge, parents focused on moving on from the critical care experiences by returning to previous activities or integrating of new care needs into their daily lives. In recognizing the differences in the lengths of interview, it is important to note that these variations correlated with the child's recovery status. Parents of children who had fully recovered tended to provide briefer accounts. This variation underlined the diverse individual circumstances of each family and contributed to a comprehensive understanding of post‐PICU recovery.

Overall, the emotional, social and transitional health experiences were shaped by the child's health trajectory, parental perception of adaptation and family dynamics. As parents attempt to re‐establish a sense of normalcy, the state of emotional health varies according to the needs and health of the child. This was consistent with the PICS‐p framework, which suggests that the trajectory of parental experiences varies with the child's recovery. Unlike other studies, our participants tended not to recall their stressful experiences during critical care admission, with its first mention only at the second interview, at 6 months after discharge (Atkins et al., 2012; Jakobsen et al., 2021; Rennick et al., 2021; Terp & Sjostrom‐Strand, 2017).

As families reintegrate back into the community, parental social capital as determinants of health should be examined. Parental social capital refers to the network of social relationships, resources and support available to parents as they navigate the post‐discharge phases after their child's critical illness (Sawyer et al., 2019). Structural support from the healthcare system was seen initially at post‐discharge and over time these interactions were reduced and were targeted to the specific needs of the survivors. Social workers and speciality nurses were important liaison between the parents and the hospital. Previous studies highlighted parental difficulties in receiving paediatric‐specific support while in the community due to the lack of expertise and issues with handover fragmentation from the tertiary to community health (Poh, Carey, et al., 2022). Continuation of informational support and a source of contact from the admitting hospital may facilitate transitions in the first 6 months of discharge.

Studies have shown that an individual's cognitive social capital and perception of available social resources are associated with the quality of life (Muckenhuber et al., 2016). Parents in the current study perceived themselves as being well supported by formal networks such as hospitals and social resources such as friends and family. Reports from parents may reflect a low level of relational social capital as shown by the inability to share their experiences with their friends and family, there was a lack of a local community for parents of children with similar conditions. Understanding the level of parental social capital can help clinicians, researchers and policymakers identify areas where additional support and interventions to enhance parental long‐term experiences after discharge from the PICU.

In parental transitional health, the changes in family dynamics were more prominent for children requiring medical care at 6 months after discharge. Mothers of children who faced challenges, with spouses' illness‐related involvement, reported more frustration. There was sparse evidence of spousal involvement and parental experiences after PICU discharge. Traditionally, the main roles of fathers were provider and protector (Clarkson & Hearn, 2021). Parenting and caregiving roles were gendered and was considered a feminine trait in many cultures (McDonnell et al., 2019). A systematic review showed that positive father's involvement in illness‐related tasks was associated with better health outcomes in neonatal intensive care survivors (Filippa et al., 2021). In one study, infants had better weight gain, sleep and psychosocial behaviours (Hearn et al., 2020). Fathers reported that early involvement in the neonatal ICU cultivated the feelings of ‘closeness’ which helped them transitioned between traditional masculine behaviours and traditional feminine practices of caring for children with health issues (Clarkson & Hearn, 2021). Although the care trajectory for paediatrics is different from the neonatal population, in which, fathers' involvement was shaped prior to the PICU admission. The involvement of fathers during admission may help to enhance ‘closeness’ and inclusivity. Previous studies conducted in the PICU reported that fathers had different psychological responses and help‐seeking behaviours and reported more post‐traumatic growth (personal strength) compared to mothers (Foster et al., 2019; Rodriguez‐Rey & Alonso‐Tapia, 2019). Fathers previously reported feelings of being marginalized in the healthcare settings, to overcome this, fathers should be involved in treatment plans and the caring needs of their child in the PICU (Taylor et al., 2020). Further research should concentrate on determining what aspects of fathers' involvement during the PICU admission were most beneficial, and its impact on long‐term parental transitional health after discharge.

### 5.1 Strengths and limitations

It is important to acknowledge that this study focused on the experiences of parents following their child's initial PICU admission. Thus, these findings may not fully encapsulate the potentially varied experiences of families dealing with recurrent PICU admissions. In additional, the presence of PICU survivors with their parents during the interviews may have limited parent's openness. Parents of these children may be influenced by their protective instinct or to exhibit socially desirable traits while sharing their challenges and the issues they have encountered with the care of the child (Polit & Beck, 2020). However, through the convenience of video conferences, we were able to interview participants during the COVID‐19 safe‐distancing restrictions and were able to interview 22 parents at both 1 and 6 months after discharge. Repeated interviews in longitudinal studies allow researchers to gather rich, detailed data about participants' experiences and changes over time. Families who did not schedule for the second interview at 6 months might be experiencing more challenges or might have moved on from the PICU admission and hence were less interested in discussing their experiences.

### 5.2 Recommendations for further research

Future research could investigate the trajectory of parental long‐term outcomes beyond the 6‐month mark. Understanding how parental emotional health evolves over time, particularly when their children returnto school or when they have achieved normalcy. The newly termed parental transitional health domain identified in this study could be further investigated by comparing the experiences of parents from different PICU settings. A broader understanding may potentially inform the development of context‐specific support interventions.

### 5.3 Implications for policy and practice

This study highlighted the critical role of parents in childhood critical illness. Providers can emphasize the importance of collaborative care models, engaging parents in the discussion about their child's care, discharge planning and transition back to normalcy to enhance the effectiveness of care delivery.

## 6 CONCLUSION

Overall, this study offers novel insights into long‐term outcomes, parental experiences and support needs, with implications for policy and practice. Moreover, it features methodological innovation through the utilization of repeated sequential semi‐structured interviews as a means to investigate long‐term parental experiences after their child's critical illness. Utilization of the PICS‐p framework as a working framework for data analysis further enhanced the credibility of the results by providing a holistic lens to analyse parental emotional and social health experiences. An additional domain, termed parental transitional health, was incorporated to underscore the complexities of the process of reintegration into the home environment. While the intensity of these impacts decreased overtime, for some families, a targeted intervention to enhance parental emotional, social and transitional health which improves parental long‐term experiences after PICU discharge is warranted.

## AUTHOR CONTRIBUTIONS

**PF, MC, JM, JH, JL**: The conception and design of the study or acquisition of data or analysis and interpretation of data. **PF, MC, JM, JH, JL:** Drafting the article or revising it critically for important intellectual content. **PF, MC, JM, JH, JL**: Final approval of the version to be submitted.

## FUNDING INFORMATION

This work was partly funded by the KKH Academic Medicine Start‐up funds of KK Women's and Children's Hospital, Singapore (KRDUK20AR100) and SingHealth FY2019 Talent Development Fund (TDF) Award‐Research. The funders were not involved in the conduct of the study.

## CONFLICT OF INTEREST STATEMENT

The authors declare no conflict of interest.

### PEER REVIEW

The peer review history for this article is available at https://www.webofscience.com/api/gateway/wos/peer‐review/10.1111/jan.16288.

## Supporting information


Appendix S1.

Appendix S2.

Appendix S3.

Data S1.

## Data Availability

The data that support the findings of this study are available from the corresponding author upon reasonable request.
