# Supplementary material for: Parental emotional, social and transitional health in the first 6 months after childhood critical illness: A longitudinal qualitative study
Source: J Adv Nurs. 2024 Jun 24;81(2):978–93. doi: 10.1111/jan.16288 (PMC11730748; doi:10.1111/jan.16288)

ESM 2. Framework analysis, stage 4, example of categories within developing a working analytical framework.


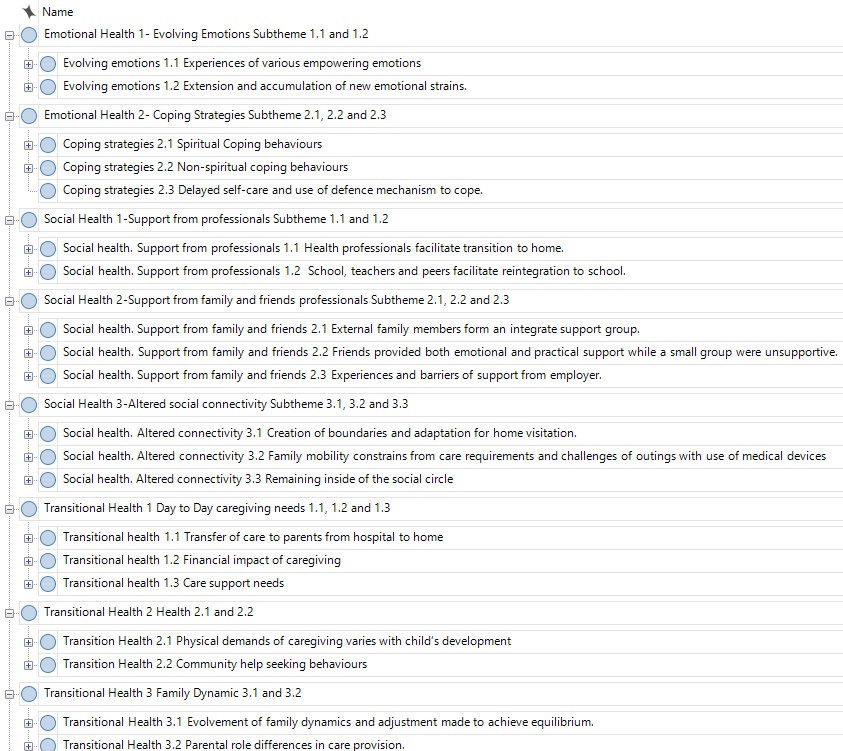

Supplement: Supplementary file 2 — Appendix S2. [file JAN-81-978-s004.docx]
