# Supplementary material for: Parental emotional, social and transitional health in the first 6 months after childhood critical illness: A longitudinal qualitative study
Source: J Adv Nurs. 2024 Jun 24;81(2):978–93. doi: 10.1111/jan.16288 (PMC11730748; doi:10.1111/jan.16288)
Supplement: Supplementary file 3 — Appendix S3. [file JAN-81-978-s003.docx]

ESM 3 Sample of data analysis: Themes, Subthemes and Quotations of Parental Emotional, Social Health and Transitional health at 1 and 6 months post PICU discharge.

| **Domain: 1. Parental Emotional Health,**  **Theme 1: Evolving Emotions**  Subtheme 1.1: Experiences of various empowering emotions | | |
| --- | --- | --- |
| **Quotations at 1 month** | **Codes within subtheme** | **Quotations at 6 months** |
| “…but ah one thing is that the medical expertise of Singapore is really something that nobody should be doubting.” (Taufiq, Malay-Mother)  “I came across so many foreigners I came across the Filipinas, the Myanmar, the Singaporean you know, they give 110% TSK dedication, I was very, I was very impressed, because you know why? for me ah. I have never been on a hospital bed ever since I was born, you know what I mean so when you accompany your daughter in the hospital for one and a half week , eh for one and a half months you can see work! this people do. I am really impressed, you know what I mean I am really impressed.” (Nadia, India-Father)  “(I am) scared you know, I am not a nurse, even injection for myself will be very very painful, I am to carry out injection at home **but I have to be brave for him, he is brave. I don’t have a choice**, I have to learn slowly then he can go home.” (Abdul, Malay-Mother)  “We have to be strong, to not affect other children. **We have to cope well, we have to cope fast so that everybody is on track so that we can get back to our normal life you know,** **try to minimise any changes for the family**. So ah.. its just that like now, now I have to wake up earlier to prepare breakfast, and lunch for him to bring to school. As a mother, this is something I have to do right, to help my kid (laughter), I mean its ok for me.” (Chris, Chinese-Mother) | Trust in the healthcare system for access to medical needs, continuation of support and medical recommendations (T1 and T2)  Being parent: the ability to overcome adversity quickly to care for child and family. (T1 and T2) | “give your 100% trust to the professionals, they know best. Don’t go and be kaypoh (nosy) "why you doing this to my son, why you not doing this way." Leave it to the professionals, and do not hinder, do not interfere with their treatment to your child. ya and another thing that I need to highlight is, regardless of the ward parents choose, your kid will get the correct treatment” (Taufiq, Malay-Mothers)  “Financially yes, I still need financial support. Because of COVID I was retrenched, I was not working since January. And as for the expenses I got some support. Even when I was in KKH, I got some financial support, they reduced the expenses for the hospitalisation.” (Niya, Indian-Father)  “nah (laughter), I gave to her so I have to bear her bad behaviours (laughter).” (Linda, Chinese-Mother)  “(laughter) its ok for me, as long as I see the weight gain then I’m very happy.” (Aish, Malay-Mother) |
