# Supplementary material for: Parental emotional, social and transitional health in the first 6 months after childhood critical illness: A longitudinal qualitative study
Source: J Adv Nurs. 2024 Jun 24;81(2):978–93. doi: 10.1111/jan.16288 (PMC11730748; doi:10.1111/jan.16288)
Supplement: Supplementary file 4 — Data S1. [file JAN-81-978-s001.docx]

COREQ table

| No | Item | Guide questions/description | Remarks |
| --- | --- | --- | --- |
| Domain 1: Research team and reflexivity --- team: 3 staff members with extensive experiences in PICU care + an intensivist. | | | |
| Personal Characteristics | |  |  |
| 1. | Interviewer/facilitator | Which author/s conducted the interview or focus group? | Pei-Fen Poh.  “the interviewer who was a female, **Singaporean, Chinese** PhD student and a paediatric critical care nurse.” Page 8, line 21. |
| 2. | Credentials | What were the researcher's credentials? E.g. PhD, MD | PhD candidate, page 8 line 21. |
| 3. | Occupation | What was their occupation at the time of the study? | PICU nurse, page 8 line 21. |
| 4. | Gender | Was the researcher male or female? | Female, page 8 line 21. |
| 5. | Experience and training | What experience or training did the researcher have? | Qualitative methods training MSc PhD, and supported by academic supervisors who are experienced qualitative researchers.  Page 8 line, 21. |
| Relationship with participants | |  |  |
| 6. | Relationship established | Was a relationship established prior to study commencement? | The researcher did not provide direct care to potential participants to avoid conflicts of interest. Page 6, line 2. |
| 7. | Participant knowledge of the interviewer | What did the participants know about the researcher? e.g. personal goals, reasons for doing the research | Participant were aware that the interviewer was a PICU nurse. Page 6, line 30. |
| 8. | Interviewer characteristics | What characteristics were reported about the interviewer/facilitator? e.g. Bias, assumptions, reasons and interests in the research topic | Page 8, line 21 |
| Domain 2: study design | |  |  |
| Theoretical framework | |  |  |
| 9. | Methodological orientation and Theory | What methodological orientation was stated to underpin the study? e.g. grounded theory, discourse analysis, ethnography, phenomenology, content analysis | Framework analysis using PICS-p as an apriori framework, page 5 line 21. |
| Participant selection | |  |  |
| 10. | Sampling | How were participants selected? E.g. purposive, convenience, consecutive, snowball | Purposive sampling recruitment method was utilised, Page 6, line 3. |
| 11. | Method of approach | How were participants approached? E.g. face-to-face, telephone, mail, email | Face-to -Face during child’s admission at child’s bedside, page 6, line 2. |
| 12. | Sample size | How many participants were in the study? | 28 and 22 parents at 1 and 6 months after PICU discharge, page 9, line 2. |
| 13. | Non-participation | How many people refused to participate or dropped out? Reasons? | 3 declined out of 31 eligible participants, page 6, line 2. |
| Setting | |  |  |
| 14. | Setting of data collection | Where was the data collected? E.g. home, clinic, workplace | Hospital, bedside, home over teleconference, page 6, line 18. |
| 15. | Presence of non-participants | Was anyone else present besides the participants and researchers? | “During data collection, it was noted that children were present at the time of the interviews in some instances.” Page 7, line 6 |
| 16. | Description of sample | What are the important characteristics of the sample? E.g. demographic data, date | Characteristics of participants on Table 2 included child’s age, gender, ethnicity, length of admission and diagnosis, page 9, line 3. |
| Data collection | |  |  |
| 17. | Interview guide | Were questions, prompts, guides provided by the authors? Was it pilot tested? | Yes, the guide was pilot tested with 3 family members for readability, page 2, line 15. |
| 18. | Repeat interviews | Were repeat interviews carried out? If yes, how many? | Yes, 1 month and 6 months post discharge, page 6, line 23. |
| 19. | Audio/visual recording | Did the research use audio or visual recording to collect the data? | All interviews were audio-recorded, page 6, line 25. |
| 20. | Field notes | Were field notes made during and/or after the interview or focus group? | Audio-recorded interviews were conducted and transcribed verbatim by PF with field notes incorporated in brackets, page 7, line 12. |
| 21. | Duration | What was the duration of the interviews or focus group? | 7 – 76 minutes, page 9, line 7. |
| 22. | Data saturation | Was data saturation discussed? | “**Throughout the data collection process, we monitored for data saturation, this was achieved after the twentieth interview. We conducted three more interviews to ensure that the data truly represent a comprehensive understanding of parental experiences after PICU discharge.” Page 6, line 26.** |
| 23. | Transcripts returned | Were transcripts returned to participants for comment and/or correction? | Members checks were conducted with study participants to validate data interpretation, page 8, line 27 |
| Domain 3: analysis and findings | |  |  |
| Data analysis | |  |  |
| 24. | Number of data coders | How many data coders coded the data? | PF coded and checked by MC and JL, page 7, line 17. |
| 25. | Description of the coding tree | Did authors provide a description of the coding tree? | A sample of the coding tree can be found in the electronic supplement 2 |
| 26. | Derivation of themes | Were themes identified in advance or derived from the data? | Page 7, line 32. |
| 27. | Software | What software, if applicable, was used to manage the data? | NVivo 12, page 7, line 19. |
| 28. | Participant checking | Did participants provide feedback on the findings? | Yes, preliminary findings were discussed with the participants, page 8, line 27. |
| Reporting | |  |  |
| 29. | Quotations presented | Were participant quotations presented to illustrate the themes / findings? Was each quotation identified? e.g. participant number | Yes |
| 30. | Data and findings consistent | Was there consistency between the data presented and the findings? | Yes |
| 31. | Clarity of major themes | Were major themes clearly presented in the findings? | Yes |
| 32. | Clarity of minor themes | Is there a description of diverse cases or discussion of minor themes? | Yes, point 4.6.0 COVID-19 restriction, page 19, line 28. |
